# Mix it and fix it: functions of composite olfactory signals in ring-tailed lemurs

**DOI:** 10.1098/rsos.160076

**Published:** 2016-04-20

**Authors:** Lydia K. Greene, Kathleen E. Grogan, Kendra N. Smyth, Christine A. Adams, Skylar A. Klager, Christine M. Drea

**Affiliations:** 1 Department of Evolutionary Anthropology, Duke University, Durham, NC, USA; 2 University Program in Ecology, Duke University, Durham, NC, USA; 3 Department of Biology, Duke University, Durham, NC, USA

**Keywords:** olfactory communication, scent mark, composite signal, strepsirrhine primate, behavioural bioassay, intrasexual competition

## Abstract

Animals communicating via scent often deposit composite signals that incorporate odorants from multiple sources; however, the function of mixing chemical signals remains understudied. We tested both a ‘multiple-messages’ and a ‘fixative’ hypothesis of composite olfactory signalling, which, respectively, posit that mixing scents functions to increase information content or prolong signal longevity. Our subjects—adult, male ring-tailed lemurs (*Lemur catta*)—have a complex scent-marking repertoire, involving volatile antebrachial (A) secretions, deposited pure or after being mixed with a squalene-rich paste exuded from brachial (B) glands. Using behavioural bioassays, we examined recipient responses to odorants collected from conspecific strangers. We concurrently presented pure A, pure B and mixed A + B secretions, in fresh or decayed conditions. Lemurs preferentially responded to mixed over pure secretions, their interest increasing and shifting over time, from sniffing and countermarking fresh mixtures, to licking and countermarking decayed mixtures. Substituting synthetic squalene (S)—a well-known fixative—for B secretions did not replicate prior results: B secretions, which contain additional chemicals that probably encode salient information, were preferred over pure S. Whereas support for the ‘multiple-messages’ hypothesis underscores the unique contribution from each of an animal's various secretions, support for the ‘fixative’ hypothesis highlights the synergistic benefits of composite signals.

## 1. Introduction

When communicating with conspecifics, animals often use composite signals that comprise elements from either multiple or single sensory modalities (including vocal, visual, olfactory and tactile modalities) [1–3]. Whereas multimodal signals include elements from minimally two modalities, unimodal composite signals include minimally two elements from the same modality [3]. Whether investigating multimodal or unimodal signals, attributing function to composite signals presents a significant challenge [4,5], because one must deconstruct the signal to assess the contribution of its individual components and also examine how those elements potentially enhance, complement or modulate one another [3]. Composite signals that incorporate olfactory elements [6–8] further exacerbate the challenge because even pure scent signals can be extremely complex and can contain hundreds of chemical compounds that might serve different functions [9,10]. We therefore specifically lack understanding about the function of composite olfactory signals. Here, using the scent-marking system of a strepsirrhine primate, we test two non-mutually exclusive hypotheses about the function of composite olfactory signals.

The conceptual framework for understanding the evolution and function of composite signals emerged primarily from the study of multimodal signals [2,3,11–13], which have been classified (or dichotomized) as fixed or fluid and as redundant or non-redundant. The elements of fixed signals (e.g. [14]) cannot be uncoupled from one another, whereas fluid signals (e.g. [15]) can vary in components, timing or sequence [3,16]. Redundant (or backup) signals are those for which the individual elements communicate identical information. For example, both the auditory and visual components of the honeybee (*Apis mellifera*) ‘waggle’ dance provide information about the distance and direction to food sources [17]. Redundancy in multimodal signalling may increase communication success or emphasis [3,12], as evidenced by the enhanced or amplified responses such signals generate in receivers [3,18–20]. By contrast, the elements of non-redundant signals convey unique information, such that combining them allows multiple messages to be contained within the same signal [11,12]. These non-redundant elements can modulate one another [3], enhance signal efficacy or produce novel information [2]. More generally, the components of multimodal signals may draw attention or prime receivers for additional information [6,21–23]. The vibrational cues of dancing honeybees, for example, alter conspecific responsiveness to other elements within the multimodal signal [22].

Much of the theoretical framework developed for multimodal signals also applies to unimodal composite signals [3,5]. For instance, to convey social dominance, mandrills (*Mandrillus sphinx*) rely on fixed, redundant visual cues, including the vibrancy of their red and blue facial markings, as well as the contrast between colours [24]. By comparison, components of human speech, including pitch, amplitude and frequency, all convey different information and, thus, represent non-redundant elements of a vocal composite signal [25]. Based on descriptive studies, numerous taxa rely on composite chemosignals [26–30]; however, in only a handful of cases has the function of odorant mixtures been examined [10,31,32]. This under-representation may owe to the fact that, of the various types of composite signals, perhaps the most challenging to decipher are those involving olfactory elements.

Although certain scent signals can be relatively simple, as is the case with many insect pheromones [33], most mammalian olfactory signals are exceptionally complex. Mammalian scent derives from diverse sources, including urine, faeces, saliva, skin secretions and specialized scent glands [9,34,35], and can be deposited alone or as mixtures. The ‘pasted’ scent marks of brown hyenas (*Parahyaena brunnea*), for instance, comprise two anal-gland secretions originating from sebaceous and apocrine tissues, respectively, but are deposited in close succession within the same marking event [36]. The resulting composite mark includes a long-lasting white paste and a more ephemeral brown paste that, in addition to their visible contrast, differ in their chemical composition [37]. The chemical compounds within any given scent signal routinely range in structure, volatility and longevity [34,35,38,39]. Information can be conveyed by single compounds [33] or classes of compounds [40], by their presence or absence, relative ratios, absolute abundances [41] or by the entire odour mosaic [42]. When odorants from two or more sources are combined, the resulting composite signal could represent information that is additive or it could represent information that differs synergistically from that of the pure secretions.

Boasting a complex scent-marking repertoire [43–46], the ring-tailed lemur (*Lemur catta*) is an appropriate model for the study of composite olfactory signals [6]. Endemic to Southwest Madagascar, ring-tailed lemurs are diurnal generalists that reside in territorial, multimale–multifemale groups [47,48]. Like most lemurs, they are characterized by female social dominance over males and by strict reproductive seasonality [49,50]. Olfactory signalling in ring-tailed lemurs is critical both for inter- and intra-group communication: both sexes scent mark [6,43,45,51] within and along territorial boundaries [52], depositing chemically rich, genital secretions that encode the signaller's sex, reproductive state and individual identity [10], as well as genetic relatedness and genetic quality [40,53–55]. Males additionally possess two sex- and species-specific scent glands, the antebrachial (A) and brachial (B) glands [56], the usage of which forms the basis of this study.

The secretions from the males' A and B glands, respectively, are highly volatile or greasy and rich in squalene (S) [10,57], which is a common component of mammalian secretions and available commercially as a fixative [58–60]. Males broadcast their secretions in various ways: ‘wrist marking’, whereby the A gland and its adjacent spur are drawn across a substrate (visibly and audibly scarring it) [43], minimally serves to deposit pure A secretions. Because A secretions can be pre-mixed with B secretions via ‘shoulder rubbing’, whereby males draw their A gland across their B gland, wrist marking also can serve to deposit a composite (A + B) signal. Alternatively, these two secretions can be mixed by males rubbing both of their glands against their tail fur (via tail marking), followed by wafting the scent-impregnated tail at conspecifics during ritualized ‘stink fights’ [43]. Curiously, B secretion is not naturally deposited alone.

Wrist marking and shoulder rubbing, whether used for depositing secretions on a substrate or for self-anointing, form part of multimodal displays that include both fixed and fluid components. Both displays always include fixed, but ephemeral, visual elements (i.e. the stereotypical postures required for scent deposition). When used for marking substrates, the display also includes fixed, but ephemeral, auditory elements (i.e. the clicking sound from the spur) and produces fixed, but long-lasting, visual elements (i.e. the permanently scarred substrate that can become a communal signpost) [6,43,51]. The fluid components of these multimodal signals derive entirely from their olfactory elements, because males can variably mix A and B secretions. Although pure A and B secretions share some chemical compounds that could potentially function as redundant signals [10], they also contain unique chemical blends that elicit different conspecific responses [10,61], indicating that A and B likely also convey non-redundant information (i.e. multiple messages).

Working within the framework of non-redundant signals, we use behavioural bioassays and a discrimination paradigm to test two, non-mutually exclusive hypotheses about the function or modulatory effect of mixing A and B secretions. According to the ‘multiple-messages’ hypothesis, if A and B secretions each encode unique information, mixing them may increase the information conveyed in a single scent mark [3,11,12], thereby increasing the interest of signal recipients. According to the ‘fixative’ hypothesis, adding viscous, long-lasting B secretions (S, in particular) to highly volatile A secretions may function to increase the longevity of A's information after deposition in the environment [10,29,34,60,62], thereby prolonging the signal's interest to recipients. Under this hypothesis, chemical elements would be non-redundant, but would not necessarily convey new information.

In a first ‘natural experiment,’ we investigate male behavioural response to pure A, to pure B and to mixed A + B secretions from conspecifics, presented concurrently in either fresh or decayed conditions. If both secretions contribute unique information, responders should always prefer mixtures over pure secretions of comparable age. If, however, B secretions (S, in particular) mainly preserve A secretions without imparting any new information, responders should prefer A and/or A + B secretions over B secretion when fresh, but only A + B secretions when decayed. In a second ‘synthetic experiment,’ we replicate the prior design, but replace B secretions with S throughout, testing if this fixative alone can reproduce the function of mixing natural secretions.

## 2. Material and methods

### 2.1. Subjects and housing

The subjects were 12 adult, reproductively intact, male ring-tailed lemurs (mean age ± s.d.: 11.1 ± 9.7 years; range: 1.6–30.5 years) that served both as secretion ‘donors’ and as behavioural bioassay ‘recipients’. The animals belonged to six mixed-sex groups housed at the Duke Lemur Center (DLC) in Durham, NC, USA. Details on animal housing, diet and reproductive seasonality in the Northern Hemisphere have been described previously [10]. We ran the study in the breeding season (November 2013–February 2014), during morning hours (8.00–12.30).

### 2.2. Animal handling and odorant collection

To facilitate odorant collection, trained DLC handlers carefully caught and manually restrained the subjects. We then gently pressed the animals' A or B glands and swabbed their secretions with cotton that had been pre-cleaned with methanol and pentane [10,61]. The DLC animals are habituated to these brief (less than 15 min) procedures and experience minimal stress. Moreover, researchers have shown that the same patterns of information content in odorants (e.g. differences by sex, age, rank) are detected whether secretions are collected directly from the animal or after the animal deposits them in the environment [63]. For the natural experiment, we mixed secretions by collecting A, then B, exudates onto the same swab. For the synthetic experiment, we collected A secretions using a swab that had been pre-dipped into a vial of S (99%, Acros Organics, Morris Plains, NJ, USA). ‘Fresh’ samples were immediately enclosed in pre-cleaned chromatography vials, which we placed on dry ice and then stored at −80°C within 2 hours. ‘Decayed’ samples were placed in pre-cleaned, uncapped vials, so as to be exposed to ambient air and were left at room temperature for 12 hours (see electronic supplementary material, S1). After this period, we capped the vials and stored them at −80°C.

### 2.3. Experimental design

During a series of 10 min bioassay trials [55,61], we presented subjects, temporarily isolated from their group, with a choice between pure and mixed odorants. For each trial, we used three samples thawed to room temperature, each rubbed onto a clean wooden dowel. We secured the dowels at a 45° angle to the enclosure fence, placing them in randomized order between left, centre and right positions [61]. In the natural experiment, each trial involved presenting A, B and A + B odorants derived from one donor unfamiliar to the recipient. Recipients participated in two cycles of two trials per day, randomized within days for the fresh and decayed conditions of a given donor's odorants, and randomized across cycles for the two different donors. The synthetic experiment, conducted a month later, was identical except that, using two new, unfamiliar donors, we presented fresh or decayed A, S and A + S odorants.

### 2.4. Data collection

All trials were videotaped and began after dowel placement. Using an established ethogram [61], an observer blind to odorant placement transcribed the recipients' responses. Scoring began once the inter-observer reliability scores exceeded 95% concordance [64], during training, between the observer and a trainer who had extensive experience scoring videos of ring-tailed lemur bioassays; all trials were then scored in random order within a three-week period. The responses recorded were readily identifiable and easily distinguished (see fig. 1 in [54]): they included investigation of the ‘scent mark’ (sniffing and licking), as well as various forms of scent marking or ‘countermarking’ (wrist marking, shoulder rubbing, genital marking, tail marking and biting of the substrate). The investigatory responses could reflect recipient interest in the odorant, as well as the degree of processing required by the signal, whereas countermarking is more purely responsive in nature.

**Figure 1.**
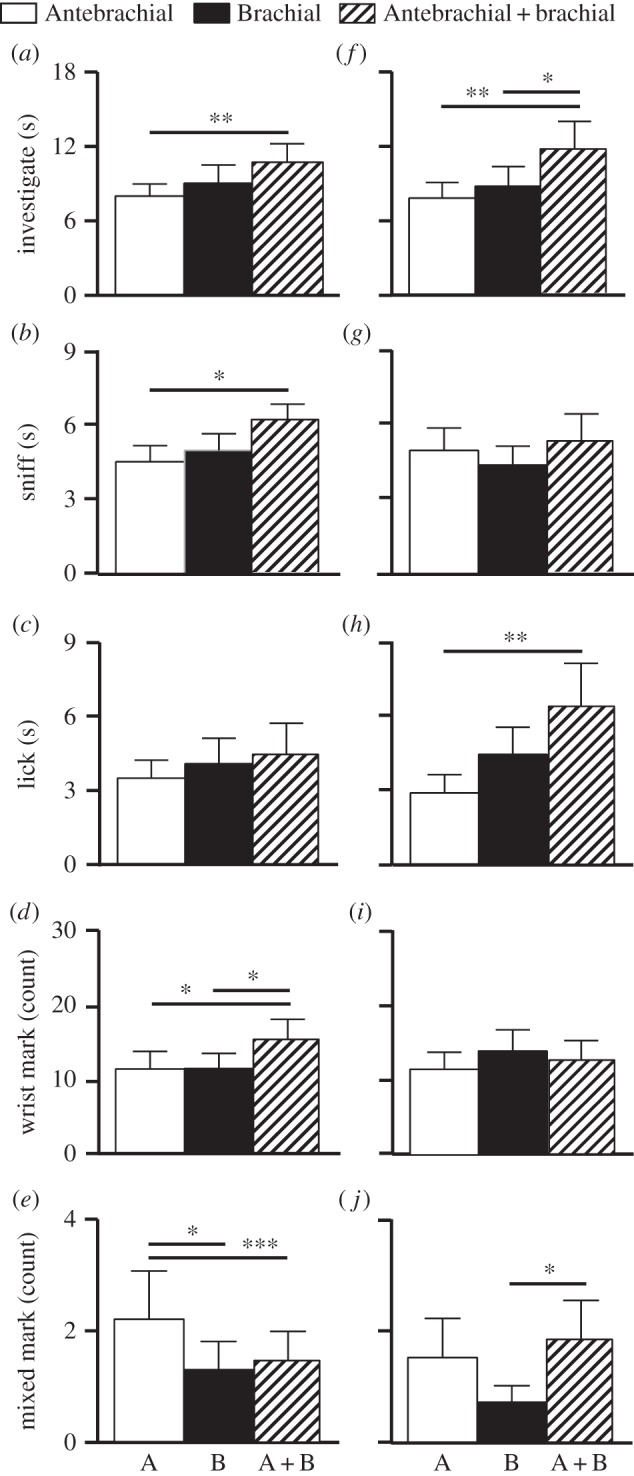
Mean + s.e.m. responses by male ring-tailed lemurs during the natural experiment to pure (A or B) and mixed (A + B) secretions, presented in fresh (*a–e*) and decayed (*f–j*) conditions. Asterisks denote significant *p*-values (*p < 0.05, **p < 0.01, ***p < 0.001).

### 2.5. Statistical analyses

We tested the influence of odorant type and freshness on recipient response using generalized linear mixed models (GLMM) that we implemented using the glmmADMB package (v. 0.7.7) in Rstudio (v. 0.98.501). We analysed the duration of sniffing and licking, as well as the duration of total investigation (sniffing + licking). Because males occasionally insert shoulder rubs (as somewhat infrequent events) within bouts of wrist marking, we analysed two measures associated with wrist marking: the total frequency of wrist marking and the frequency of wrist marking that occurred within 1 min of shoulder rubbing (as a proxy of A + B countermarks, hereafter termed ‘mixed marking’). Of the various forms of countermarking, only wrist marking and its subset of mixed marking occurred at frequencies sufficient for statistical analysis (genital marking, tail marking and biting the substrate were directed at less than 1%, 7% and 9% of the presented odorants, respectively).

We ran a single GLMM for each condition (fresh versus decayed) and each experiment (natural versus synthetic). Responses were entered as individual data points; the dependent variable was behavioural response and the independent variable was odorant type (A, B, A + B or A, S, A + S). We included trial number (first or second) on a given test day, and donor identity nested under each recipient, as random terms. We used the Poisson distribution or negative binomial distribution in cases when the data were overly dispersed and we used AIC values to select the best-fit distribution [65]. When appropriate, we also applied a zero-inflation correction factor [65]. Lastly, to compare the responses to B and S odorants across all trials, we used a single GLMM, as above, except that we used odorant type (B or S) and condition (fresh or decayed) as independent variables.

## 3. Results

As predicted by the multiple-messages hypothesis, male ring-tailed lemurs responding to conspecific scent in the natural experiment generally attended to the mixed secretions more than to either of the pure secretions. Notably, during the fresh condition, males investigated A + B significantly more than A (*z* = 2.88, *p* < 0.01; figure 1*a*). This result was driven primarily by the males' sniffing behaviour, which was directed at A + B significantly more than at A (*z* = 2.42, *p* < 0.02; figure 1*b*). Males showed no difference, however, between licking any of the fresh secretions, whether mixed or pure (figure 1*c*). Males also wrist marked in response to fresh A + B significantly more than they did to either A (*z* = 2.48, *p* < 0.02) or B (*z* = 2.03, *p* < 0.05; figure 1*d*). The main exception to the general preference for mixed over pure secretions, when fresh, was in mixed marking (figure 1*e*): when considering only the subset of wrist marks that were closely preceded by shoulder rubbing, males marked significantly more in response to pure A secretions than to A + B mixtures (*z* = 3.99, *p* < 0.001; figure 1*e*). Moreover, as predicted by the fixative hypothesis, males performed significantly more mixed marking in response to fresh A than to fresh B (*z* = 2.55, *p* < 0.02; figure 1*e*).

As predicted by both hypotheses, male interest in mixed over pure secretions was even stronger in the decayed condition; however, the lemurs modified their manner of investigation and countermarking. Notably, the males investigated A + B significantly more than either A (*z* = 2.79, *p* < 0.006) or B (*z* = 2.07, *p* < 0.04; figure 1*f*), but in contrast to the fresh condition, it was the males' licking behaviour that primarily drove this finding: whereas the males showed no difference in sniffing the decayed mixture over either of the pure secretions (figure 1*g*), they licked decayed A + B significantly more than A (*z* = 2.83, *p* < 0.005; figure 1*h*). Unlike with fresh odorants, males showed no differences in total wrist-marking frequency in response to decayed mixtures compared to pure odorants (figure 1*i*). Nevertheless, males deposited significantly more mixed marks in response to A + B secretions than to pure B secretions (*z* = 2.00, *p* < 0.05; figure 1*j*).

**Figure 2.**
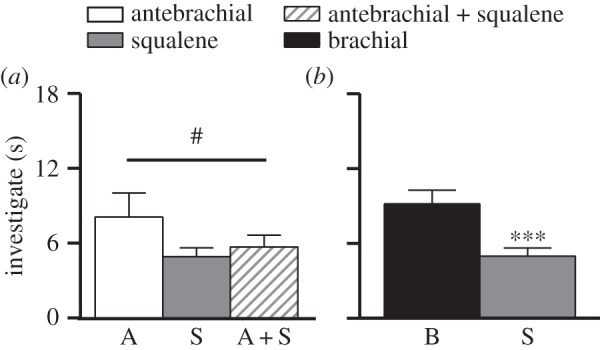
Mean + s.e.m. responses by male ring-tailed lemurs during the synthetic experiment. Responses to squalene (S) were low in the fresh condition (*a*) and when compared to B secretions across odorant ages (*b*). # denotes a trend at *p* < 0.10, *** denotes significance at *p* < 0.001.

Contrary to expectations, the findings from the synthetic experiment did not replicate those of the natural experiment. Instead, lemurs responded the least to S, whether pure or mixed, fresh or decayed. Although the males investigated A modestly more than A + S, in both the fresh (*z* = 1.62, *p* = 0.10; figure 2*a*) and decayed (*z* = 1.77, *p* < 0.08, data not shown) conditions, there were no differences between any of the odorants, whether fresh or decayed, in the males' distribution of sniffing, licking, wrist marking or mixed-marking behaviour (data not shown). Lastly, compared to their earlier responses to B (in the natural experiment), subjects investigated S significantly less (*z* = −3.35, *p* < 0.001; figure 2*b*) and tended to wrist mark less in response to S (*z* = −1.75, *p* = 0.08; data not shown).

## 4. Discussion

In this study of conspecific response to composite olfactory signals, focused on the male ring-tailed lemur, we provide new evidence to support minimally two functions of scent mixing. Consistent with the ‘multiple-messages' hypothesis [3,12], a mixture of two secretions often generated more responsiveness (or interest) than did either of the pure secretions alone. Recipient responses revealed that composite chemical signals probably function to increase the number of messages contained within a single scent mark. That recipient preference for the mixture strengthened after the secretions had decayed also supports the ‘fixative’ hypothesis [10,29,34,62], suggesting that, beyond contributing unique chemical information, lemur B secretions help preserve lemur A secretions. This latter interpretation is also supported by the pattern of recipient mixed countermarking to fresh and decayed signals. Although squalene, a prevalent component of B secretions, might be a major fixing agent, the recipient males overwhelmingly preferred natural secretions, regardless of their freshness, to those mixed with synthetic squalene. Perhaps at the ‘dosage’ used, pure squalene became somewhat aversive. Based on these results, we suggest that the mixing of low- and high-molecular weight compounds, respectively, contained in lemur A and B secretions, serves dual functions, notably increasing the information content of composite scent marks while also prolonging their longevity. Thus, the incorporation of multiple communicatory elements in unimodal (and even multimodal) signals may serve several, concurrent functions, with synergistic benefits.

Support for the ‘multiple-messages' hypothesis might have been anticipated from the distinct chemical properties of the pure forms of the lemurs' two secretions [10] and the different responses they generated [61]. Given that males can deposit either pure A secretions or a mixture of A and B secretions [6,43], these signals would be classified as both fluid and non-redundant [3,16]. That being the case, it is unclear why male lemurs do not naturally deposit pure B secretions, particularly given that they are not physically prohibited from doing so. Perhaps unlike A secretions, that function in pure or mixed form, the unique messages contained in B secretions lack meaning or relevance if uncoupled from the messages contained in A secretions. Therefore, particularly as suggested by the mixed countermarking of respondents, the primary role of B secretions appears to be to modulate A secretions.

Beyond providing additive (A + B) messages, the synergistic benefit of combining these chemical elements also owes, presumably, to the properties of squalene, in support of the ‘fixative’ hypothesis. Glandular tissue specialized in the production (or expression) of certain low-molecular-weight compounds (as evidenced by the A organs) may lack the versatility necessary to also produce specific high-molecular-weight compounds (and vice versa). The dramatic differences between the morphology of A and B glands [56] and the chemical composition of their secretions [10] extend well beyond B's contribution of squalene. Perhaps synergism also derives from the additional modification of compounds in mixed signals. With growing appreciation for the role of bacterial fermentation in odorant production [34,66] and decay [39], we now recognize that unique communities of obligate or facultative anaerobic microbes inhabit warm, moist scent glands and produce many of the chemical compounds (e.g. volatile fatty acids: [67]) used by their hosts in olfactory signalling [68]. Once glandular secretions are deposited, the bacteria shed may be exposed to different light, oxygen, temperature or pH conditions that could influence their action. Likewise, new microbes could be introduced from the substrate marked, altering the structure of the microbial communities within the deposited mark. Continued microbial action over the lifetime of a deposited secretion could be responsible for signal longevity or specific patterns of decay [39]. Lastly, the blending of multiple scent-gland microbiomes and their signature chemicals may underlie the modulation of chemical information within composite olfactory signals.

Regarding the perception of odorants by signal recipients, most mammals have two distinct sets of chemosensory neurons (located in the olfactory epithelium and in the vomeronasal organ) that, respectively, process small, volatile chemicals versus larger, fluid-phase molecules [69,70]. The male lemurs' preferential sniffing (over licking) of fresh mixtures is consistent with the processing of ephemeral, volatile information via the olfactory epithelium. Their preferential licking (over sniffing) of decayed mixtures is consistent with the processing of enduring, non-volatile information [69,70] via their functional vomeronasal organ [71]. Licking may also function to moisten dehydrated secretions and release conserved volatile information.

Regarding scent-marking responses following odorant perception, countermarking is a common response to deposited odorants that can produce olfactory collages, often positioned along territorial boundaries (e.g. [52,72–78]). The various functions proposed for countermarking [79] include delineating territorial boundaries, mediating intrasexual competition, advertising reproductive state and converging on a group signature. Male ring-tailed lemurs routinely overmark the odorants of both sexes, potentially indicating that countermarking serves both competitive and reproductive functions [6,45]. Our present results are consistent with the competitive function of countermarking, but also illustrate how both the age and composition of the signal may further modulate the recipient's response. Notably, fresh, but not decayed, mixtures, elicited the most recipient wrist marking, potentially reflecting perception of (and response to) a ‘nearby’ intruder male. Once decayed, the mixtures may no longer represent the same imminent threat. In a similar manner, mixed countermarks (or the subset of wrist marks that were preceded by shoulder rubs) were most frequent in respondents encountering fresh A or presumably preserved A (i.e. decayed A + B), perhaps suggesting that the greatest threats elicit the most enduring competitive response. The inclusion of B secretions into scent countermarks could allow male respondents to either alter the messages encoded (according to the ‘multiple-messages’ hypothesis) or to alter the intended audience (according to the ‘fixative’ hypothesis). More broadly, short-lived signals may be directed at nearby conspecifics or group members, whereas enduring signals may be directed at non-group members that may eventually visit the composite marks. Thus, to mix or not to mix secretions in response to competitive odorants may depend on the concurrent inter- and intra-group social contexts [2], as inferred by the qualitative features of the marks encountered.

Wrist marking occurred far more frequently than did shoulder rubbing, perhaps because the greasy secretions from a single shoulder rub are sufficient to affect multiple wrist marks. The scarcity with which we observed genital marking in this study may have owed to our focus on male response to male odour: because genital secretions contain salient information about mate quality [53–55], genital marking may be more relevant to intersexual signalling than to competitive, intrasexual signalling. Tail marking, which occurs during ritualized ‘stink fights' between males [43], may have been infrequently observed in this study because it requires the physical presence, rather than the mere olfactory presence, of a competitor.

The incorporation of unimodal variation within multimodal signals is not unique to ring-tailed lemurs. Many species create composite olfactory signals by combining scents from various sources [73,80,81]. Mammalian chemical signals are often also multimodal, incorporating short-term [7,36,78,82–86] or enduring [87–89] visual aspects (associated with the deposition of odorants in a particular manner or locale) that draw the attention of nearby conspecifics [3,6]. Nevertheless, our understanding of the flexibility with which, or the context in which, mammals include or exclude elements remains limited. Future studies could profitably address the chemical consequences of odorant mixing, the decay of individual and composite odorants, the chemical modulation of signals resulting from repeated overmarking by single or multiple individuals and the bacterial mechanisms underlying host chemical communication. Understanding conspecific response to temporal and compositional variation likewise will be key for assessing signal function. By presenting evidence that mixing olfactory signals can minimally serve two functions—increasing information and prolonging messages—we hope to spur additional comparative investigations into the function of composite olfactory communication.

## Supplementary Material

ESM1. Determining decay conditions and Figure S1.

## Supplementary Material

ESM2. Greene, Grogan et al. data.

## Data accessibility

The data are provided as electronic supplementary material, S2.
