# Supplementary material for: Mix it and fix it: functions of composite olfactory signals in ring-tailed lemurs
Source: R Soc Open Sci. 2016 Apr 20;3(4):160076. doi: 10.1098/rsos.160076 (PMC4852645; doi:10.1098/rsos.160076)
Supplement: ESM1. Determining decay conditions and Figure S1. [file rsos160076supp1.docx]

**ELECTRONIC SUPPLEMENTARY MATERIAL 1**

***Determining decay conditions***

Our set up for determining adequate decay conditions for antebrachial (A) secretions, specifically, was identical to the set up we used for trials described in the main text. Briefly, from each of 3, gently restrained, male ring-tailed lemurs, we collected 6 samples of A secretions by rubbing pre-cleaned cotton swabs against the males’ A organs. We placed these samples in pre-cleaned chromatography vials: we immediately capped two sets of odorant vials and placed them at -80 ºC post collection, whereas we left the remaining 4 sets of odorant vials uncapped, exposing them to ambient temperatures for 3, 6, 12, or 24 hours. After the respective, allotted decay times, we capped the samples and also placed them at -80 ºC.

We presented these odorants, of various ‘ages,’ from each male ‘donor’ to an unrelated and unfamiliar, conspecific male ‘recipient’ during 2 separate bioassay trials that occurred on subsequent days. In all trials, we presented the males with 3 wooden dowels, each of which we rubbed with an odorant-containing swab. In one trial, the recipients received the fresh, 3-hr, and 6-hr conditions, whereas in the other trial, they received the fresh, 12-hr, and 24-hr condition, all derived from the same donor. We videotaped all of the bioassays, which a single observer scored for relevant behaviour, including total investigation, sniffing, licking, and wrist marking (as described in the main study).

Based on the behavioural responses of the recipients (Figure S1), which generally first decreased after 12-hr of air-exposure, we selected the 12-hr time point for our ‘decay’ condition in subsequent bioassay trials (presented in the main text).

**Figure S1.** Mean + s.e.m. responses by male ring-tailed lemurs to conspecific antebrachial secretions during the decay experiment. The secretions presented were fresh (black bars), or allowed to decay at room temperature for 3 hours (dark grey bars), 6 hours (grey bars), 12 hours (light grey bars), and 24 hours (white bars).
